# A quick scoping review of the first year of vaccination against the COVID-19 pandemic: Do we need more shots or time?

**DOI:** 10.1097/MD.0000000000030609

**Published:** 2022-09-16

**Authors:** Ayman El-Menyar, Naushad Ahmad Khan, Ahammed Mekkodathil, Sandro Rizoli, Rafael Consunji, Eman Elmenyar, Sagar Galwankar, Hassan Al-Thani

**Affiliations:** a Department of Surgery, Trauma and Vascular Surgery Clinical Research, Hamad Medical Corporation, Qatar; b Clinical Medicine, Weill Cornell Medical College, Doha, Qatar; c Department of Surgery, Trauma Surgery, Hamad Medical Corporation, Qatar; d Bahcesehir University, Istanbul, Turkey; e Department of Emergency Medicine, Sarasota Memorial Hospital, Sarasota, FL.

**Keywords:** booster dose, COVID-19, immunogenicity, safety, SARS-CoV-2, vaccination, viral variants of concern

## Abstract

The emergence of new severe acute respiratory syndrome coronavirus 2 variants, along with the waning of vaccine-induced immunity, has increased breakthrough infections and urged booster jabs and debates. In the short term, the administration of booster doses has been reported to be safe and enhance severe acute respiratory syndrome coronavirus 2–specific neutralizing antibody levels. However, the effects of these doses on the pandemic trajectory and herd immunity are unclear. There is insufficient evidence that a third booster shot of the coronavirus disease 2019 (COVID-19) vaccine maintains longer immunity and covers new viral variants. The lack of sufficient evidence, combined with the fact that millions of people have not yet received 1 or 2 jabs of the COVID-19 vaccine, has raised concerns regarding the call for booster vaccinations.

**Methods:**

We conducted a quick scoping review to explore the literature on the need for a booster COVID-19 vaccination from January 1, 2021, to April 30, 2022.

**Results:**

Sixty-one relevant publications were identified, of which 17 were related to waning immunity after 2 doses of the vaccine among the general population or healthcare workers, 19 were related to the third or booster dose of vaccination after the second dose among the general population or healthcare workers, and 25 were related to booster dose among immunocompromised patient.

**Conclusions:**

Initially, the need for a booster dose was equivocal; however, several studies demonstrated the benefit of the booster dose over time. Adequate scientific information is required regarding the administration of booster doses to the general population as well as the high-risk individuals.

## 1. Introduction

As the world enters the third year of the coronavirus pandemic, vaccination remains the most effective method for preventing infection and symptom severity. However, the long-term evidence of protection against severe acute respiratory syndrome coronavirus 2 (SARS-CoV-2) infection remains inconclusive.[1] In conjunction with other public health mitigation strategies such as testing and masks, vaccination is crucial for a comprehensive coronavirus disease 2019 (COVID-19) control and management approach as the pandemic evolves toward endemicity.[2] The global effect of the COVID-19 pandemic is profound, and the rate of development of COVID-19 vaccinations has been at unprecedented speed producing 1.5 billion doses per month.[3] Multiple safe and highly effective vaccines have been approved for use worldwide for less than a year after SARS-CoV-2 was identified as the causative agent of the COVID-19 pandemic.

Furthermore, the resurgence of breakthrough SARS-CoV-2 infections in developed countries with advanced vaccine programs has raised concerns regarding the effectiveness of these vaccines.^[4–7]^ Consequently, there have been arguments regarding the need for booster doses, which may be premature owing to a lack of substantial evidence of benefits.^[8]^ Calling an unplanned third dose of vaccination, especially 1 year before the first dose administration, has caused more confusion among the scientific and general communities. Existing data lack evidence of a dose–response relationship between the frequency of vaccination and the potential occurrence of complications, such as thromboembolic^[9,10]^ or serious reactive inflammatory diseases.^[11]^ Furthermore, the appropriate layman’s language to adequately explain the need for this third dose through traditional means and social media is lacking.^[12]^

To date, several countries have been unable to adequately deliver the first or second vaccine dose, raising inequities and safety concerns. Moreover, if the third or fourth dose becomes mandatory, it will expose recipients to uncertain risks of unproven long-term benefits, mostly in high-income countries.^[13]^ Moreover, in countries with the rate of 2-dose vaccination exceeding 50% to 85% of their populations, the reinfection rate with the new COVID-19 variant has increased at a rate similar to the prevaccination era with less severity and mortality.^[14]^

Herein, we sought to explore debates and arguments to determine whether there was an underestimation or overestimation of the call for more vaccination shots. This article also reviews and addresses critical unanswered questions, such as the potential need for modified vaccine formulations due to emerging viral variants to identify immune correlates of protection and the public health challenges of using various tools to combat breakthrough infections, including boosters, in an era of global vaccine shortages.

## 2. Methods

### Study design and search strategy

This quick scoping review explores the evidence for the safety, immunogenicity, and requirement of a booster COVID-19 vaccine dose after receiving the 2 primary doses. The PubMed/MEDLINE and Google Scholar databases were searched for relevant articles published in the English language in the duration between January 1, 2021, and April 30, 2022. The search terms used were (“third” OR “three” OR “booster”) AND “dose*” AND (“COVID-19” OR “SARS-CoV-2”) AND “vaccine*”. Preprint repositories, such as BioRxiv and MedRxiv, were also searched for unpublished results. This review adhered to the Preferred Reporting Items for Systematic Reviews and Meta-Analyses Extension for Scoping Reviews checklist.

### Ethical approval

It was not required for this review.

### Inclusion and exclusion criteria and study selection

To identify eligible studies published across the entire year 2021, we developed a search strategy using medical subject headings and text words for the general concepts of measures, interventions, and patients. The search results were manually reviewed, and duplicates were removed. First, the study population included the general population or healthcare workers who received 2 doses of COVID-19 vaccination, and their antibody responses measured, or breakthrough infections estimated, were included to describe waning immunity after a long follow-up (mostly 6 months). Second, the study population included the general population or healthcare workers who received a third or booster dose of vaccination after 2 doses (homologous or heterologous) to describe the safety and immunogenicity of the vaccine. Finally, immunocompromised patients who received booster doses were included to evaluate the safety and immunogenicity of the vaccine in terms of antibody levels and adverse events.

Animal studies were excluded from analyses. Publications in languages other than English, abstracts, documents, books, review articles, case reports, commentaries, or editorials were excluded. Letters to the editor reporting retrospective or prospective observational studies and clinical trials were included.

### Data extraction and quality assessment

For each review, titles, abstracts, and full texts were independently screened for inclusion according to the preestablished criteria. Disagreements were resolved via discussion; however, no specific quality-assessment tool was used. The data were extracted by 3 reviewers (A.M., E.A., and N.A.K.).

### Data processing and analysis

A narrative synthesis was performed to include numerical summaries, textual commentaries, and tabular and graphical representations. Although we aimed to include publications of 2021 only, we used some of 2022 to comment, support, or dispute our hypothesis (the need for more time or shots) for more transparency.

## 3. Results

**Figure 1.**
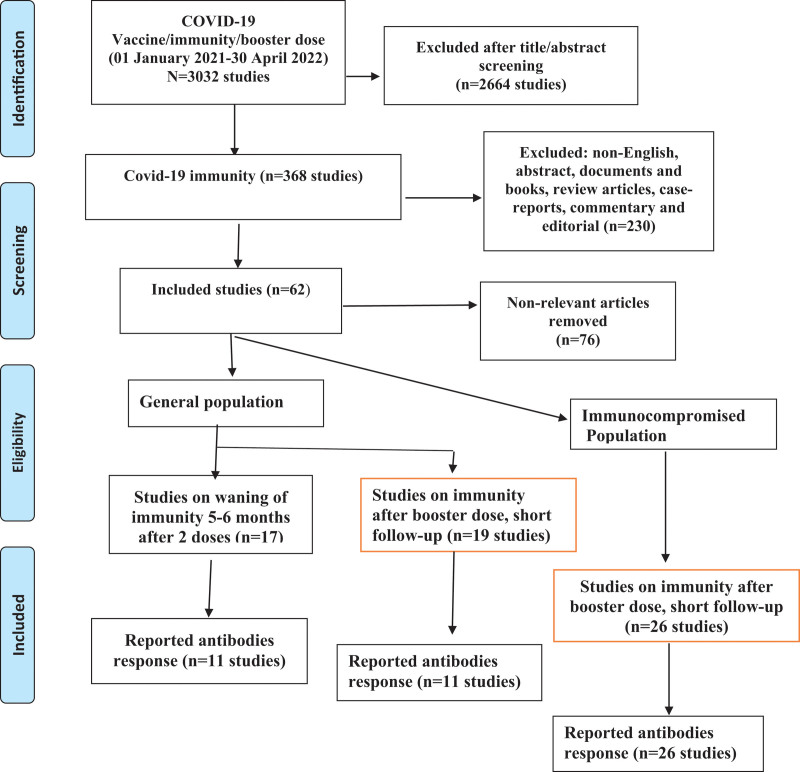
Study flowchart. COVID-19 = coronavirus disease 2019.

The search process identified 62 relevant studies: 17 were related to waning immunity, mostly after 6 months of vaccination[15–32] (Table 1 and Fig. 1), 19 papers were related to the third or booster dose vaccination (Table 2),[33–50] and 26 studies on the third or booster dose vaccination among immunocompromised patient populations (Table 3).[51–76]

**Table 1:** Summary of the studies on the waning of immunity over the time after COVID-19 vaccination.

Authors/publication year (country)	Study design/population	Objectives	Vaccine status	Methods/results	Antibody response measured	Comments
Ponticelli et al, 2021 (Italy)^[15]^	Longitudinal observational study among healthcare workers (n = 444)	Evaluate the response to BNT162b2 mRNA COVID-19 vaccine.	Two doses of BNT162b2 mRNA vaccine 21 d apart.	A quantitative serology test for research on SARS-CoV-2 S-RBD-specific IgG. More than 75% reported adverse events.	Yes	The effective innate and adaptive immune response might make women more prone to adverse events following vaccine administration. A strong seroconversion was observed based on 30-d serology, with persistence of anti–SARS-CoV-2 S-RBD IgG at 6 mo from vaccination. However, the level of vaccine-induced antibodies started to decrease from the second month.
Goldberg et al, 2021 (Israel)^[16]^	Retrospective study in general population, not been infected before the study period (n = 4791,398)	Determine the effects of the BNT162b2 vaccine on the spread of SARS-CoV-2 infection and severity of COVID-19 and the waning of vaccine protection over time.	Fully vaccinated are individuals for whom 7 d or more had passed since the second dose of the BNT162b2 vaccine.	The rate of infection between July 11 and 31 was higher among persons who became fully vaccinated in January 2021 than among those fully vaccinated 2 mo later, in March (rate ratio, 1.6), for persons aged ≥60 yr.	No	The extent of waning in the months immediately after vaccination was not quantified. Immunity against the Delta variant of SARS-CoV-2 waned in all age groups a few months after the second dose of vaccine.
Levin et al, 2021 (Israel)^[17]^	Longitudinal prospective study involving vaccinated healthcare workers (n = 4868)	Assess the dynamics of antibody levels and determine the predictors of antibody levels at 6 mo.	Participants were tested monthly for the presence of antispike IgG and neutralizing antibodies over a 6-mo period after receipt of the second dose of the BNT162b2 vaccine.	The level of IgG antibodies decreased at a consistent rate, whereas the neutralizing antibody level decreased rapidly for the first 3 mo with a relatively slow decrease thereafter.	Yes	6 mo after the second dose of the BNT162b2 vaccine, the humoral response was substantially decreased, especially among men, persons aged ≥65 yr, and persons with immunosuppression.
Lustig et al, 2021 (Israel)^[18]^	Prospective, single-center, longitudinal cohort study among healthcare workers (n = 2607)	Determine the early antibody responses and antibody kinetics after each vaccine dose in subjects of different ages and sexes, and with different comorbidities.	4026 serum samples from 2607 eligible, vaccinated participants. This study appears to be from the same center and involved the same participants as the study by Levin et al.^[17]^	Lower antibody concentrations were consistently associated with male sex ratio of means 0.84, older age (ie, ≥66 yr; 0.64), immunosuppression (0.44), and other specific comorbidities: diabetes (0.88), hypertension (0.90), heart disease (0.86), and autoimmune diseases (0.82).	Yes	The second vaccine dose is particularly important for older and immunosuppressed persons, highlighting the need for timely second vaccinations and potentially a revaluation of the long gap between doses in some countries.
Tartof et al, 2021 (USA)^[19]^	Retrospective cohort study using electronic health records of individuals who were members of the healthcare organization (n = 3,436,957)	Determine the overall and variant-specific effectiveness of BNT162b2 against SARS-CoV-2 infections and COVID-19-related hospital admissions by time since vaccination for up to 6 mo.	Full vaccination with BNT162b2, defined as receiving 2 doses of BNT162b2 within 7 d or more after the second dose.	Effectiveness against infections declined from 88% during the first month after full vaccination to 47% after 5 mo. Vaccine effectiveness against hospital admissions for infections with the Delta variant for all ages was high overall (93%) up to 6 mo.	No	The effectiveness of the vaccine against hospital admissions up until 6 mo after being fully vaccinated. Reduction in vaccine effectiveness over time might be primarily due to waning immunity with time rather than the Delta variant escaping vaccine protection (funded by Pfizer).
Bayart et al, 2021 (Belgium)^[20]^	Multicenter, prospective, and interventional study among healthcare workers (n = 231)	Conduct an interim analysis on the data obtained on the humoral response after a 6-mo follow-up.	Participants received the 2-dose regimen of BNT162b2.	At day 180, a significant antibody reduction was observed in seronegative and seropositive persons. The estimated half-life of IgG from the peak humoral response was 21 and 53 d in seronegative and seropositive persons, respectively.	Yes	A highly significant decrease in neutralizing antibodies, IgG, and total antibodies in both seropositive and seronegative persons at 6 mo after the administration of the first dose of BNT162b2.
Chemaitelly et al, 2021 (Qatar)^[21]^	Retrospective matched test-negative, case–control study based on nationwide data. People with at least 1 dose: BNT162b2 (n = 947,035); mRNA-1273 (n = 564,196); 2-dose BNT162b2 (n = 907,763); mRNA-1273 (n = 494,859)	Estimate vaccine effectiveness against any SARS-CoV-2 infection and any severe, critical, or fatal case of COVID-19.	A total of 8203 SARS-CoV-2 BNT162b2 breakthrough infections were found in persons who received 1 dose of the vaccine, and 10,543 such infections had been reported in persons who received 2 doses.	Vaccine effectiveness against any SARS-CoV-2 infection increased to 36.8% in the third week after the first dose, reached its peak at 77.5% in the first month after the second dose, and gradually declined thereafter, with the decline accelerating after the fourth month to reach approximately 20% in months 5 to 7 after the second dose.	No	Vaccine-induced protection against hospitalization and death persisted with hardly any waning for 6 mo after the second dose. Large proportion of the vaccinated population could lose its protection against infection in the coming months.
Tang et al, 2021 (Qatar)^[22]^	Retrospective study based on nationwide data. People with at least 1 dose: BNT162b2 (n = 950,232); mRNA-1273 (n = 564,468); 2-dose BNT162b2 (n = 916,290); mRNA-1273 (n = 509,322)	To assess the real-world effectiveness of COVID-19 mRNA vaccines against infection with the Delta variant.	≥14 d after vaccine (BNT162b2), (mRNA-1273), and (BNT162b2 or mRNA-1273).	≥14 d, the effectiveness against Delta infection after only 1 dose was 45.3% for BNT162b2 and 73.7% for mRNA-1273. The effectiveness after the second dose was 51.9% for BNT162b2 and 73.1% for mRNA-1273.	No	Effectiveness against any Delta-induced severe, critical, or fatal COVID-19 disease or more days after the second dose was 93.4% for BNT162b2 and 96.1% for mRNA-1273. There was evident effectiveness for BNT162b2 and mRNA-1273 at preventing Delta hospitalization and death, and lower effectiveness at preventing infection, particularly with the BNT162b2 vaccine.
Thomas et al, 2021 (multicenter, multinational)^[23]^	Placebo-controlled, observer-blinded, multinational, pivotal efficacy trial in general population (n = 44,165 age ≥ 16 yr and n = 2264 age 12–15 yr)	Determine vaccine efficacy against laboratory-confirmed COVID-19 and safety for 6 mo post vaccination.	Participants received 2-30-μg doses, at 21 d apart, of BNT162b2 or placebo.	Vaccine efficacy against severe COVID-19 disease was 96.7%. Vaccine efficacy of 86 to 100% was observed across countries, different ages, sexes, ethnic groups, and risk factors.	No	Systemic events were mostly mild to moderate in severity, but occasional severe events occurred. Safety monitoring will continue according to the protocol for 2 yrs after the second dose. A gradual decline in vaccine efficacy was found (Funded by Pfizer).
Doria-Rose et al, 2021 (USA)^[24]^	Phase 3, open-label trial including 33 healthy adults, who received 2 vaccinations (100 µg), 28 d apart, with mRNA-1273 (Moderna) stratified by age (18–55 yr, 56 to 70 yr, or >71 yr)	Determine the durability of mRNA -1273-vaccine protection against SARS-CoV-2 after 6 mo.	Two doses of mRNA-1273 (Spikevax; Moderna) vaccine 28 d apart.	Binding antibody responses to the spike receptor-binding domain were assessed by enzyme-linked immunosorbent assay. Neutralizing activity was assessed assay PsVNA assay and by live wild-type SARS-CoV-2 PRNT assay. Antibodies that were elicited by mRNA-1273 persisted through 6 mo after the second dose.	Yes	Antibody activity remained high in all age groups at day 209. Binding antibodies had geometric mean end-point titers (GMTs) of 92,451 in participants 18–55 yr of age, 62,424, in those 56 to 70 yr of age, and 49,373 in those 71 yr of age or older. Nearly all participants had detectable activity in a pseudovirus neutralization assay.
Andrew et al, 2021 (UK)^[25]^	A test-negative case–control retrospective- observational study, which included 1,659,513 participants from general population	To estimate vaccine effectiveness against symptomatic disease, hospitalization and mortality by age, comorbidity status, and over time after the second dose to investigate waning separately for Alpha and Delta variants.	Two doses of BNT162b2 mRNA vaccine, Vaxzevria and Spikevax.	A decline in vaccine protection over time; persons, who had received 2 doses of the Pfizer vaccine. Vaccine effectiveness against symptomatic infection decreased after 20 wk for Comirnaty, Vaxzevria, and Spikevax following the administration of second dose.	No	Vaccine effectiveness against symptomatic disease peaked in the early weeks after the second dose and then fell to 47.3 and 69.7 by 20+ wk against the Delta variant for Vaxzevria and Comirnaty, respectively. Vaccine effectiveness fell less against hospitalizations to 77.0 and 92.7 beyond 20 wk postvaccination and 78.7 and 90.4 against death for Vaxzevria and Comirnaty, respectively. Greater waning was observed among >65-yr-olds with underlying medical conditions compared to healthy persons.
Kertes et al, 2021 (Israel)^[26]^	Retrospective study among general population based on Maccabi Health care services database (n = 8395)	To determine if the BNT162b2 vaccine had become less effective in preventing infection, and if so, in which population groups and to what degree after 6 mo post second dose.	Individuals received the 2-dose regimen of BNT162b2.	PCR testing and measurement of IgG antibody levels of the vaccinated population over time. Serology was found to decrease over time from a mean of 14,008 for 123 those tested within a month of being vaccinated to a mean of 1411 for those tested in the sixth month after vaccination. Of all those that were vaccinated with both doses, 2.8% also had a positive PCR result.	Yes	Serology levels of participants aged ≥60 yr was almost half compared to participants under the age of 60 yr in the first month, attenuating to a <10% difference 6 mo later. Large differences in initial serology levels were also observed for participants with chronic illness (immunosuppressive disorder, renal, or heart disease).
Zhong et al, 2021 (USA)^[27]^	Longitudinal cohort study using a convenience sample of healthcare workers (n = 1960)	To examine SARS-CoV-2 spike IgG antibodies and comparing antibody durability in individuals.	Individuals received second dose of mRNA SARS-CoV-2 with or without prior SARS-CoV-2 infection.	Compared with participants without previous SARS-CoV-2 infection, those with prior infection maintained higher postvaccination adjusted median antibody measurements by an absolute difference of 2.56 (95% CI, 1.66–4.08) (relative difference, 56% [95% CI, 35%–94%]) at 6 mo.	Yes	Healthcare workers with prior SARS-CoV-2 infection followed by 2 doses of mRNA vaccine (3 independent exposures to spike antigen) developed higher spike antibody measurements than individuals with vaccination alone.
Adachi et al 2022 (Japan)^[28]^	Retrospective analysis of COVID-19 patients (n = 32).	To investigate antispike protein antibody titer at the time of breakthrough infection of SARS-CoV-2 Omicron.	Individuals infected with SARS-CoV-2 Omicron after 2 doses of the mRNA vaccine diagnosed by RT-PCR using nasopharyngeal swabs or saliva sample.	The median number of months from the second vaccination to the breakthrough infection was 5 mo (range: 2–7 mo). The median antibody titer at breakthrough infection was 776 AU/mL (IQR: 411–1805) overall, of which the median antibody titer of BNT162b2 vaccinated was 633 AU/mL (IQR: 400–994) and that of mRNA-1273 vaccinated was 9416 AU/mL (IQR: 7470–16,671).	Yes	Study suggests that breakthrough infection may occur with a higher antispike antibody titer after vaccination with mRNA-1273.
Tarke et al, 2022 (USA)^[29]^	Cross-sectional study among vaccinated adults (n = 96)	To understand the impact of more recent variants on memory T cells and B cells compared with early variants, particularly in the context of COVID-19 vaccination and evaluation of the adaptive responses induced by different vaccine platforms.	Subjects vaccinated with mRNA-1273, BNT162b2, Ad26.COV2.S, or NVX-CoV2373.	Significant overall decreases were observed for memory B cells and neutralizing antibodies. In subjects ~6 mo postvaccination, 90% (CD4+) and 87% (CD8+) of memory T-cell responses were preserved against variants on average by AIM assay, and 84% (CD4+) and 85% (CD8+) preserved against Omicron.	Yes	Omicron RBD memory B-cell recognition was substantially reduced to 42% compared with other variants. T-cell epitope repertoire analysis revealed a median of 11 and 10 spike epitopes recognized by CD4+ and CD8+ T cells, with average preservation >80% for Omicron.
Yalçin et al, 2022 (Turkey)^[30]^	Prospective observational study among healthcare workers (n = 148)	To evaluate the antibody levels after inactivated virus vaccination among healthcare workers.	Healthcare workers (74 with prior COVID-19 infection and 74 with not) received 2 doses of inactivated virus vaccine included (CoronaVac).	Serum samples were prospectively collected 3 times (days 0, 28, 56). Antibody levels after the first vaccine were very low in participants without previous COVID-19, a high titer was observed in antibody levels after the second dose.	Yes	Among those who had previous COVID-19 infection, antibody titers after the first dose of vaccine were found to be close to the titers obtained after the second dose in the previously not infected group.
Yigit et al, 2022 (Turkey)^[31]^	Retrospective study among healthcare workers (n = 678)	To determine the seroconversion rate of the CoronaVac vaccine among healthcare workers 2 mo after the second dose.	Healthcare workers administered 2 doses of CoronaVac, and with no previous history of SARS-CoV-2 infection.	Of the total, 22.9% were seronegative. Young age associated with high level of anti-SARS-CoV-2 IgG (r = −0.312, P < .001).	Yes	Anti–SARS-CoV-2 IgG levels were much higher in women than men.

AIM = activation induced marker, CI = confidence interval, COVID-19 = coronavirus disease 2019, GMT = geometric mean titer, IgG = immunoglobulins G, IQR = interquartile range, mRNA = messenger ribonucleic acid, PCR = polymerase chain reaction, PRNT = plaque reduction neutralization test, PsVNA = pseudovirus neutralization assay, RBD = receptor-binding domain, RT-PCR = reverse transcription–polymerase chain reaction, SARS-CoV-2 = severe acute respiratory syndrome coronavirus 2, S-RBD = receptor-binding domain.

**Table 2:** Summary of the studies on the third or booster doses against SARS-CoV-2 infection

Authors/publication year (country)	Study design/population	Objectives	Vaccine status	Methods/results	Antibody response measured	Comments
Falsey et al, 2021 (USA)^[32]^	Randomized, placebo-controlled, observer-blind study in healthy individuals ≥12 y (n = 11)	To assess the effectiveness of the third dose of BNT162b2 vaccine against severe disease, hospitalization, and death due to waning immunity over time.	Fully vaccinated individuals who received 2-doses of BNT162b2 vaccine 7–8 mo before.	A booster shot induced substantially higher neutralizing antibody titers against the wild type, Beta and Delta virus variants, compared to levels reported after the second dose of vaccine.	Yes	Neutralization antibody titers increased >5 times in 18–55 yr and 7 times in 65–85 yr against wild-type virus. Fifteen times high in 18–55 yr and >65–85 yr against Beta variants and >5 times high in 18–55-yr and 12 times in 65–85 yr against Delta variants.
Choi et al, 2021 (USA)^[33]^	Open-label phase 2 clinical trial on participants received 2-dose mRNA-1273 vaccine approximately 6 mo earlier (n = 80)	To evaluate safety and immunogenicity of a single booster dose of mRNA-1273 or variant-modified mRNAs, including multivalent mRNA-1273.211.	20 participants received 2 injections of 100-μg mRNA-1273 completed the blinded phase and received a single booster dose of 50-μg mRNA-1273 were selected for preliminary analysis.	Neutralizing activity measured before and after booster dose. Interim analysis showed that the mRNA-1273 booster and variant-modified boosters were safe and well tolerated.	Yes	All boosters, numerically increased neutralization titers against the wild-type D614G virus compared to peak titers against wild-type D614G measured 1 mo after the primary series; significant increases were observed for mRNA-1273 and mRNA-1273.211. All boosters increased neutralization titers against key VOCs and VOIs, including B.1.351, P.1., and B.1.617.2.
Bar-On et al, 2021 (Israel)^[34]^	Prospective cohort of older adults aged ≥60 yr received 2 doses of BNT162B2 at least 5 mo earlier (n = 1,137,804)	To evaluate and quantify the real-world rates of confirmed infection and severe illness among participants.	All patients vaccinated twice with BNT162b2 vaccine had no documented positive result on PCR	Booster group was compared with those who received only 2 vaccine doses. The rate of confirmed infection was lower in the booster group than in the nonbooster group by a factor of 11.3.	No	The rate of severe illness was lower in the booster group than in the nonbooster group by a factor of 19.5
Patalon et al, 2021 (Israel)^[35]^	Retrospective case–control study among healthcare workers aged ≥40 yr (n = 306,710)	To evaluate the initial short-term additional benefit of a 3-dose vs a 2-dose regimen against infection of SARS-CoV-2.	Full vaccination with BNT162b2, or those who received booster dose, and not having a positive PCR test prior to the follow-up period.	86% reduction in the odds of testing positive for SARS-CoV-2 after booster dose.	No	Positive test results inbooster dose group was 1.1%, while nonbooster was 6.6%, respectively.
Barda et al, 2021 (Israel & USA)^[36]^	Prospective observational 1:1 matched control study included individuals aged 12 yr and above (n = 1,158,269)	To evaluate the effectiveness of a third dose of the BNT162b2 mRNA vaccine for preventing severe COVID-19 outcomes.	Full vaccination with BNT162b2, receiving 2 doses of BNT162b2 or those who received booster dose.	Vaccine effectiveness evaluated at least 7 d after receipt of the third dose, compared with receiving only 2 doses at least 5 mo ago, was estimated to be 93% (231 events for 2 doses vs 29 events for 3 doses for admission to hospital).	No	Vaccine effectiveness for severe disease 92% in 2 doses vs 3 doses (157 vs 17 events), and 81% (44 vs 07 events) for COVID-19-related death.
Bar-On et al, 2021 (Israel)^[37]^	Prospective cohort study among general population aged ≥16 yr (n = 4,696,865)	To estimate rates of confirmed infection and severe illness among participants received booster dose and compare with those who had received only 2 vaccine doses.	Full vaccination with BNT162b2, receiving 2 doses of BNT162b2 at least 5 mo earlier or those who received booster dose.	The rate of confirmed infection was lower in the booster group than in the nonbooster group by a factor of approximately 10 and was lower in the booster group than in the early postbooster group by a factor of 4.9 to 10.8.	No	The adjusted rate difference ranged from 57.0 to 89.5 infections per 100,000 person-days in the primary analysis 34.4 to 38.3 in the secondary analysis.
Arbel et al, 2021 (Israel)^[38]^	Prospective cohort study among individuals aged ≥50 y (n = 758,118)	To estimate mortality associated with the use of the BNT162b2 booster.	Subjects received 2 doses of BNT162b2 5 mo earlier or those who received booster dose.	COVID-19 mortality among booster vs nonbooster groups compared. Mortality rate in the booster group was 0.16 per 100,000 persons/day while in the nonbooster group was 2.98 per 100,000 persons/day.	No	People who received a booster at least 5 mo after a second dose had 90% lower mortality due to COVID-19 than participants who did not receive a booster.
Yue et al, 2021 (China)^[39]^	Prospective study involving healthy volunteers participating in the development and production of inactivated vaccines (n = 355)	To evaluate the durability and effectiveness of inactivated vaccines following administration of 2 doses.	Full vaccination with inactivated vaccine, receiving 2 doses or those who received third or booster dose.	Measurement of neutralizing antibodies titers in the convalescent sera of COVID-19 patients following booster dose of inactivated vaccine. At 1 mo after the second dose, the positive conversion rate of serum neutralizing antibodies reached 88.5%. However, at 8 mo after the second dose, the serum neutralizing antibody titers in this cohort decreased significantly, and the positive conversion rate decreased to 48.5%.	Yes	The positive conversion rate of antibodies increased to 95.5% after 1 mo of the third dose. Three doses of vaccine showed a more neutralizing antibody response than 2 doses of the inactivated SARS-CoV-2. Vaccine.
Berec et al, 2021 (Czech)^[40]^	Retrospective study based on nationwide data (n = 10,701,777)	To estimate the extent of the waning of postvaccination and postinfection immunity against SARS-CoV-2 infections, COVID-19 hospital admissions, and deaths and to assess the influence of the type of vaccine and previous PCR-confirmed SARS-CoV-2 infection.	Full vaccinated individuals, receiving 2 doses of vaccines or those who received booster dose.	A booster dose was shown to restore the vaccine effectiveness back to the levels seen soon after the completion of the basic vaccination schedule. The use of a booster dose returns the protection to or above the estimates in the first 2 mo after the second dose.	No	The vaccine effectiveness against hospital admissions and deaths reduced at a significantly lower rate. The postinfection immunity decreases over time.
Yang et al, 2021 (China)^[41]^	2 randomized, double-blind, placebo-controlled, phase 1 and 2 trials among adult (18–59 yr) general population (n = 40 + 450)	To assess the safety and immunogenicity of protein subunit vaccine ZF2001 and determine the appropriate dose and schedule for an efficacy study.	ZF2001 third dose after 1 mo of second dose.	Vaccination with the 25 or 50 μg doses and 2 dose or 3 dose schedules was well tolerated.	Yes	Frequency of adverse events between the vaccine and placebo groups was similar in both phase 1 and phase 2.
Saciuk et al, 2021 (Israel)^[42]^	Retrospective study among general population (n = 947,131)	To determine the vaccine effectiveness of a third dose of BNT162b2 vaccine against SARS-CoV-2 infection. Subjects who had no evidence of infection prior to day 7 postvaccination of last dose and up to the start of the study period were included.	Full vaccinated individuals, receiving 2 doses of vaccines or those who received booster dose.	The study groups were those who received 2 doses and were at least 7 d post–second vaccination and those who received third dose and were at least 7 d postvaccination. Crude Vaccine effectiveness was 92.9%, and adjusted rate was 89.1%.	No	The third dose provides added protection against SARS-CoV-2 infection for those vaccinated 6 mo ago.
Flaxman et al, 2021 (UK)^[43]^	RCT among adult general population (substudy of COV001 and COV002 RCT) (n = 75)	To assess response to booster dose given 28–38 wk after second dose.	ChAdOx1 nCoV-19 vaccine vs meningococcal conjugate vaccine (MenACWY) control.	Antibody titers after 28 d postbooster dose was significantly higher than 28 d after second dose.	Yes	Reactogenicity following late second or booster dose was lower than reactogenicity after first dose.
Eliakim-Raz et al, 2021 (Israel)^[44]^	Retrospective study among those aged ≥60 yr (n = 97)	To assess anti-S IgG antibody titers before and after a third dose (booster) of BNT162b2.	3 doses of BNT162b2 in older adults without previous COVID-19 infection and active malignancy.	The median titer level increased significantly following booster dose, from of 440 to 25,468 AU/mL (*P* < .001), and all participants became seropositive.	Yes	No major SAEs were reported.
Atmar et al, 2022 (USA)^[45]^	An open-label, nonrandomized, adaptive-design clinical trial (n = 458) conducted in sequential stages at 10 sites in United States	To study immunogenicity and safety profile of homologous and heterologous booster vaccines.	Fully vaccinated adults (≥18 yr) receive one of 3 vaccines as booster dose.	Trial included 3 vaccines such as mRNA-1273, Ad26.COV2.S, BNT162b2 as third or booster dose, and therefore provided a possibility of 9 different combinations of primary vaccination and booster in the study.	Yes	Injection-site pain, malaise, headache, or myalgia were very common. Heterologous boosters increased neutralizing antibody titers by a factor of 6 to 73, while homologous boosters increased by a factor of 4 to 20. Spike-specific T-cell responses increased in all except in homologous Ad26.COV2.S-boosted subgroup.
Suah et al, 2022 (Malaysia)^[46]^	Test-negative study among adult general population based on national administrative data (n = 1,921,403)	Comparison of homologous and heterologous booster effectiveness for CoronaVac and AZD1222 primary vaccination recipients under Delta and Omicron dominance.	Primary vaccinated and “boosted” adult population (≥18 yr).	Vaccination:3 × BNT162b2;2 × CoronaVac + AZD1222;2 × CoronaVac + BNT162b2;3 × CoronaVac2 × AZD1222 + BNT162b2;3 × AZD1222;2 × CoronaVac;2 × AZD1222;2 × BNT162b2.	No	Homologous CoronaVac and AZD1222 boosting are less effective than heterologous boosting and homologous BNT162b2 boosting.
Romero-Ibarguengoitia et al, 2022 (Mexico)^[47]^	Prospective study among general population (n = 17)	To evaluate the effect of booster dose of BNT162b2 in individuals after a completed Ad5-nCoV vaccination regimen.	To compare SARS-CoV-2 spike 1 to 2 IgG antibody titers after immunization with Ad5-nCoV, and after combining Ad5-nCoV and BNT162b2.	Those immunized with heterologous vaccine protocol had higher antibody titers and no serious adverse events when vaccines were applied 90 d apart. In addition, patients with a previous SARS-CoV-2 infection history had more elevated antibody titers as well.	Yes	Patients received BNT162b2 after Ad5-nCoV had higher SARS-CoV-2 spike 1 to 2 IgG antibody titers and had no severe adverse reactions.
Sheng et al, 2022 (Taiwan)^[48]^	Prospective study among general population (n = 399)	To compare the immunogenicity and safety of heterologous ChAdOx1/mRNA-1273 vaccination versus standard homologous ChAdOx1/ChAdOx1 and mRNA-1273/mRNA-1273 vaccination.	Four groups of prime-boost vaccination: Group 1, ChAdOx1/ChAdOx1 8 wk apart; Group 2, ChAdOx1/mRNA-1273 8 wk apart; Group 3, ChAdOx1/mRNA-1273 4 wk apart; and Group 4, mRNA-1273/mRNA-1273 4 wk apart.	On day 28 after the second dose, the anti-SARS-CoV-2 IgG titers of both heterologous vaccinations (Group 2 and Group 3) were significantly higher than that of homologous ChAdOx1 vaccination (Group 1), and comparable with homologous mRNA-1273 vaccination (Group 4). The heterologous vaccination group had better neutralizing antibody responses against the Alpha and Delta variant as compared to the homologous ChAdOx1 group.	Yes	Most of the adverse events were mild and transient. AEs were less frequent when heterologous boosting was done at 8 wk rather than at 4 wk.
Edara et al, 2022 (USA)^[49]^	Cross-sectional study among general population; 2–4 wk post primary series (n = 24); post 6 mo (n = 25), and 1–4 wk after a third dose (n = 52), and a COVID-19-recovered then mRNA-vaccinated cohort (6 mo after second dose; n = 37).	To measure neutralization activity against Omicron in a live-virus assay.	Primary series by BNT162b2 or mRNA-1273 vaccines.	Found majority of the subjects lost detectable neutralizing antibody titers against Omicron after 6 mo after the primary series (initial 2 doses) of mRNA vaccination.	Yes	The data suggest that a third booster dose is necessary to sustain neutralizing activity against Omicron.
Keskin et al, 2022 (Turkey)^[50]^	Cross-sectional study among healthcare workers and healthy controls (n = 68)	To determine IgG-S, and IgG-N of SARS-CoV-2 titers to investigate the interplay between humoral immune responses.	2 doses of CoronaVac vaccine and third dose either CoronaVac or BNT162b2. Participants with no history of COVID-19 included.	IgG-S titers were substantially higher in 2 CoronaVac + BNT group than 3 CoronaVac and control group. Conversely, median IgG-N titers were higher in 3-IVV group than other groups.	Yes	Third CoronaVac inoculations yield 1.7 and 1.8 times increases in median values of IgG-S and IgG-N titers, respectively; BNT162b2 administration as the third vaccine dose boosted IgG-S median titers by a factor of 46.6, but IgG-N titers decreased by a factor of 6.5.

3-IVV = three times coronaVac vaccinated-inactivated whole virus vaccine, AE = adverse event, COVID-19 = coronavirus disease 2019, IgG = immunoglobulins G, PCR = polymerase chain reaction, RCT = randomized controlled trial, SAE = severe adverse event, SARS-CoV-2 = severe acute respiratory syndrome coronavirus 2, VOC = variants of concern, VOI = variants of interest.

**Table 3:** Summary of the studies on booster dose of COVID-19 vaccine among immunocompromised patients.

Authors/publication year (country)	Study design/population	Objectives	Vaccine status	Methods/results	Antibody response measured	Comments
Davidovic et al, 2021 (Austria)^[51]^	Prospective cohort of chronic hemodialysis patients (n = 41)	To determine the antibody response over time during a 6-mo follow-up after vaccination.	All patients vaccinated twice with BNT162b2 vaccine.	Antibody response was determined after first injection, on the day of the second, day 28, and day 180 after the second vaccine dose by quantifying IgG antibodies in patient serum.	Yes	The cellular immune response data were lacking, including vaccine-induced T-cell response, which was found in 62%–78% of hemodialysis patients 3 to 8 wk after vaccination with BNT162b2.
Peled et al, 2021 (Israel)^[52]^	Prospective cohort of adult heart transplant patients (n = 96)	To evaluate the safety and immunogenicity of third homologous dose of the BNT162b2 vaccine. Vaccine-induced antibody responses of both RBD IgG and neutralizing antibodies were assessed.	Third homologous dose of the BNT162b2 vaccine at 168 d after the second dose.	At 18 d post third dose, the positive antibody response increased from 23% to 67%, with a simultaneous increase in neutralizing capacity. The third dose caused SARS-CoV-2 neutralization titers >9-fold and IgG anti-RBD antibodies >3-fold of the range observed after the 2 initial doses.	Yes	A homologous booster dose of BNT162b2 vaccine provides overall consistent tolerability and a good safety profile (after 18 d).
Benotmane et al, 2021 (France) (n = 159)^[53]^	Retrospective study in kidney transplant recipients (n = 159)	To measure antibody responses of third dose of the mRNA-1273 vaccine.	3 doses of mRNA-1273 vaccine. Third dose after 1 mo of second dose.	Third dose induced a serologic response in 49% of transplant recipients who did not respond after 2 doses.	Yes	Patients had weak response after the second dose were more likely to develop an antibody response after third dose compared with those without an antibody response.
Massa et al, 2021 (France)^[54]^	Prospective longitudinal study in kidney transplant recipients (n = 61)	To assess immunogenicity of 3 doses of vaccine.	3 doses of the BNT162b2 vaccine.	Spike-specific IgG seroconversion raised from 44.3% after the second dose to 62·3% after third dose (*P* <.05). Serum neutralizing activity increased after third dose for all variants of concern.	Yes	The frequency of spike-specific IFN-γ-secreting cells increased from 19.9 to 64.0 cells/million PBMCs after the third dose (*P* < .0001).
Bertrand et al, 2021 (France)^[55]^	Retrospective study in kidney transplant recipients (n = 80)	To measure antibody and T-cell responses of third dose BNT162b2 vaccine.	3 doses of the BNT162b2 vaccine. Third dose after 1 mo of second dose.	Third dose increases the rate of positive antibody and T-cell responses in nonresponsive patients after second dose and improves the magnitude of these responses in already seropositive patients.	Yes	Vaccine appears safe in terms of acute rejection and de novo donor-specific antibodies up to 1 mo postbooster dose.
Chavarot et al, 2021 (France)^[56]^	Retrospective in kidney transplant recipients (n = 62)	To determine humoral response in belatacept-treated kidney transplant recipients.	3 doses of the BNT162b2 vaccine with no history of COVID-19.	Only 6.4% of patients developed anti-SARS-CoV-2 IgG with low antibody titers (median 209, IQR [20–409] AU/mL).	Yes	Third dose of BNT162b2 vaccine did not improve immunogenicity in patients treated with belatacept without prior COVID-19.
Dekervel et al, 2021 (France)^[57]^	Prospective cohort study in hemodialysis patients (2 cohorts: n = 66 and 34)	To determine humoral response to the third dose in 2 cohorts of hemodialysis patients.	3 doses of the BNT162b2 vaccine. Third dose after 1 mo of second dose.	Anti-S IgG was found in 83.3% and 92.4% of patients after second and third doses, respectively.	Yes	Humoral response was boosted after third dose, allowing seroconversion in more than half of nonresponders.
Masset et al, 2021 (France)^[58]^	Retrospective study in kidney and pancreas transplant recipients (n = 136)	To assess humoral response of vaccine in kidney and pancreas transplant recipients.	3 doses of the BNT162b2 vaccine with no history of COVID-19.	Third dose improved humoral response from about 50% to 70%, reducing the negative impact of antimetabolite drugs and steroids on seroconversion.	Yes	Third dose largely improved intensity of humoral response, reaching titers suggestive of neutralizing antibody activity.
Westhoff et al, 2021 (Germany)^[59]^	Retrospective study in kidney transplant recipients (n = 10)	To assess humoral and cellular immune response of third vaccine dose in primary nonresponders.	3 doses of the BNT162b2 vaccine in which nonresponse reported for 2 previous doses.	Third dose elicited a humoral and cellular response in 60% and 90%, respectively, in whom primary vaccination failed.	Yes	Frequencies of cytokine-producing T cells and follicular T-helper cells increased, showing a gain of antiviral functionality.
Hall et al, 2021 (Canada)^[60]^	Randomized study (vaccine vs placebo) among transplant patients (n = 120)	To assess serologic response of vaccine in transplant patients.	Third dose of mRNA-1273 or saline placebo after 2 mo of second dose. No history of COVID-19.	The third dose in transplant recipients had substantially higher immunogenicity than placebo.	Yes	The trial had short follow-up and was not powered to detect differences in clinical outcomes.
Reischig et al, 2021 (Czech)^[61]^	Retrospective and prospective cohort study in kidney transplant recipients. Kidney transplant recipients (n = 226) vs unvaccinated patients (n = 194)	To measure IgG levels in the cohort after vaccination (n = 31) and recovery from COVID-19 (n = 19).	2 doses of BNT162b2 vaccine.	Enzyme-linked immunosorbent spot assay performed. Short posttransplant periods were associated with COVID-19 after vaccination (*P* < .001). Only 16% of patients achieved positive SARS-CoV-2 IgG after vaccination, and 89% (*P* < .001) recovered from COVID-19 (median IgG levels, 0.6 vs 52.5 AU/ml, *P* < .001).	Yes	Severity of infection; need for hospitalization; and mortality were comparable between study groups. However, short posttransplant periods and less positive cases associated with vaccination.
Redjoul et al, 2021 (France)^[62]^	Retrospective study in recipients of allogeneic hematopoietic stem cell transplantation (n = 42)	To report the humoral response to vaccine.	3 doses of the BNT162b2 vaccine.	Third dose led to a significant increase in IgG (S-RBD) from 737 to 11 099 AU/mL. However, only 48% reached the protective threshold of ≥4160 AU/mL.	Yes	B-cell count >0.25 g/L in peripheral blood at the time of third vaccination (*P* = .0032) and an IgG (S-RBD) concentration of >1000 AU/mL after second dose were associated with the rise to the protective antibody threshold.
Tillmann et al, 2021 (Germany)^[63]^	Prospective cohort study (95 chronic hemodialysis patients and 60 controls)	To determine the effect of vaccination in hemodialysis patients, search for risk factors for non- or low response, and to measure the effect of booster dose in non- or low responders.	Patients with vaccination failure were offered a third booster dosage (n = 10).	Booster dose induced an increase in effective antibody titers of >30 AU/mL in 70% patients 4–5 wk later.	Yes	The main risk factors for vaccination failure are older age and immunosuppressive therapy.
Shroff et al, 2021 (USA)^[64]^	Phase 1 trial in cancer patients (n = 20)	To compare immune responses to third dose of vaccine.	3 doses of the BNT162b2 vaccine.	At 1 wk after third dose, 16 patients had threefold increase in neutralizing antibody responses, but no improvement in T-cell responses.	Yes	Mild adverse events.
Werbel et al, 2021 (USA)^[65]^	Retrospective study among recipients of solid organ transplants (n = 30)	To describe antibody responses and vaccine reactions who received third dose due to suboptimal response to standard vaccination.	Ad26.COV2.S; mRNA-1273; BNT162b2 vaccine.	Antibody titers increased after third dose in one-third of patients who had negative antibody titers, and, in all patients, who had low-positive antibody titers.	Yes	Antibody responses, appear to vary, and potential risks, such as organ rejection, should be evaluated on an individual basis.
Marlet et al, 2021 (France)^[66]^	Retrospective study in kidney transplant recipients (n = 160) and chronic lymphocytic leukemia patients (n = 20)	To characterize antibody responses induced by a third dose of mRNA vaccines.	3 doses of BNT162b2 or mRNA-1273.	A moderate increase in antispike IgG levels observed in 12 patients with kidney transplant after the third dose (0.19 vs 5.28 BAU/mL, *P* = .03).	Yes	In 20 leukemia patients, a moderate increase in antispike IgG levels was observed in longitudinal follow-up after the third dose (0.63 vs 10.7 BAU/mL, *P* = .0002).
Karaba et al, 2022 (USA)^[67]^	Retrospective study among solid organ transplant recipients (n = 47) and healthy controls (n = 15)	To measure antispike IgG, pseudo neutralization, and live-virus neutralization against VOCs before and after a third vaccine dose in comparison with healthy controls after 2 vaccine doses.	Transplant patients with 3 doses of vaccine (70% mRNA, 30% Ad26.COV2.S) compared with 15 healthy controls after 2 doses of mRNA.	Booster vaccine dose increased median total antispike (1.6-fold), pseudo neutralization against VOCs (2.5-fold vs Delta), and neutralizing antibodies (1.4-fold against Delta).	Yes	Neutralization activity was significantly lower than healthy controls (*P* < .001); 32% of patients had zero detectable NAb against Delta post booster dose compared to 100% for controls.
Jurdi et al, 2022 (USA)^[68]^	Prospective, multicenter cohort study among kidney transplant recipients (n = 51)	To measure antiviral antibody responses against wild type and variants of SARS-CoV-2.	3 doses of BNT162b2 or mRNA-1273.	Diminished antibody response against Omicron variant was evident after third dose of mRNA vaccine in kidney transplant recipients. No patients developed allograft injury, de novo donor-specific antibodies or allograft rejection.	Yes	Breakthrough infection (6%) occurred at a median of 89 d.
Heinzel et al, 2022 (Germany)^[69]^	Single-center, single-blinded, 1:1 randomized, controlled trial among kidney transplant recipients (n = 169)	To assess changes in antibody response following a third vaccination with mRNA or vector vaccine in kidney transplant recipients from month 1 to month 3 after vaccination.	mRNA (BNT162b2 or mRNA-1273) or vector (Ad26COVS1) as third dose vaccine.	Overall seroconversion rate at 3 mo following third vaccination was comparable. However, heterologous third booster vaccination with Ad26COVS1 resulted in significantly higher antibody levels in responders.	Yes	Significantly higher number of individuals with antibody levels above predefined antibody thresholds associated with neutralizing capacity.
Abravanel et al, 2022 (France)^[70]^	Prospective study among belatacept-treated solid organ transplant patients (n = 68)	To assess humoral and cellular immune responses of belatacept-treated solid organ transplant patients to 3 doses of mRNA-based vaccine.	Three doses of BNT162b2 vaccine given between 2 doses of belatacept, ie, 2 wk after the last belatacept infusion.	Total antibodies to SARS-CoV-2 spike protein were assessed. Patients produce low humoral and cellular responses to 3 doses of BNT162b2 vaccine.	Yes	Only 23.5% of patients developed a detectable antispike response.
Chauhan et al, 2022 (USA)^[71]^	Prospective study in liver transplant recipients and chronic liver disease patients (45 liver transplant and 35 liver disease)	To assess antibody response associated with mRNA or JnJ vaccines. Poor Ab response defined as “undetectable” if Ab levels are ≤0.80 U/mL and “low” if levels are between 0.80 and 249.9 U/mL.	Two doses of mRNA vaccines or a single dose of JnJ vaccine.	After the booster dose, 73% had good response 28% had poor response.	Yes	No patient had any serious adverse events.
Gounant et al, 2022 (France)^[72]^	Prospective study in cancer patients (n = 306)	To assess humoral responses to vaccine in patients with thoracic cancer.	BNT162b2 vaccine; 2 doses: 283 patients and 3 doses: 30 patients.	Booster dose given to 1% of patients with persistent low antibody titers resulted in an 88% immunization rate	Yes	Most of the patients with thoracic cancer immunized after 2 doses.
Del Bello et al, 2022 (France)^[73]^	Retrospective study in solid organ transplant patients (n = 396)	To determine humoral response to vaccine in solid organ transplant patients.	3 doses of the BNT162b2 vaccine. First 2 doses 1 mo apart and third 59 d after second dose.	The prevalence of anti–SARS-CoV-2 antibodies was 1.3%, 5.1%, 41.4%, and 67.9% before first, second, third doses, and 4 wk after third dose, respectively.	Yes	No SAE or acute rejection episode reported after third dose.
Bensouna et al, 2022 (France)^[74]^	Prospective case series in patients treated with hemodialysis or peritoneal dialysis (n = 69)	To measure humoral response after 3 doses of the BNT162b2 vaccine in patients.	3 doses of the BNT162b2 vaccine. Third dose after 1 mo of second dose.	Spike protein S1 immunoglobulin measured after the second dose and at least 3 wk after the third dose of the BNT162b2 vaccine, which resulted in substantial increase of antibody levels.	Yes	Adverse events did not seem to be more common or severe after a third vaccine dose.
Le Bourgeois et al, 2022 (France)^[75]^	Retrospective study among allogeneic hematopoietic stem cell transplant recipients (n = 102) and healthy controls (n = 25).	To assess antibody response associated with third dose of BNT162b2 in allotransplanted patients.	Allotransplanted + 3 doses of BNT162b2 (n = 80); allotransplanted + no 3 doses (n = 22); healthy + no 3 doses (n = 25).	Booster dose increases the humoral response and antibody levels.	Yes	Neither COVID-19 infection nor graft-versus-host disease reactivation was reported.
Robert et al, 2022 (France)^[76]^	Prospective study among chronic hemodialysis patients (n = 18).	To report serological response at day 28 after receiving vaccine.	3 doses of the BNT162b2 vaccine.	Antispike IgG titer decreased with time in not only naive but also COVID-19 patients. So, the robustness of response vaccine in hemodialysis patients is certainly weaker than in the general population.	Yes	Only 50% of the full protected patients at dose 1, in terms of neutralizing antibodies, conserved this status at dose 3.

Ab = antibody, COVID-19 = coronavirus disease 2019, IgG = immunoglobulins G, IQR = interquartile range, mRNA = messenger ribonucleic acid, NAb = neutralizing antibody, RBD = receptor-binding domain, SAE = severe adverse event, SARS-CoV-2 = severe acute respiratory syndrome coronavirus 2, VOC = variant of concern.

Waning of immunity studies were among fully vaccinated, that is, 2 doses of vaccine, mostly the BNT162b2 mRNA vaccine, and the immune response was measured postvaccination.^[15–32]^ Evidence of immunity and its decline in the general population was evaluated by measuring the antibody response in terms of immunoglobulin (Ig)G antibody levels^[15,17,18,20,24,26–31]^ or clinical evidence of breakthrough infection.^[16,19,21–23]^ The maximum follow-up duration was 6 months.^[15–32]^ Postvaccination adverse events have been reported in a few studies and only for a short postvaccination follow-up period. Immune response was evident starting at 2 weeks postvaccination and up to 6 months postvaccination. Subsequently, the immune response wans.^[15–32]^

Antibody responses after third or booster dose vaccination among the general population^[32,33,39,41,43–45,47–50]^ and among immunocompromised patients^[51–76]^ have also been evaluated in multiple studies. The waning of immunity against different variants of SARS-CoV-2, such as Delta^[16,19,22,25]^ and Omicron,^[28,29]^ has been reported in several studies. In contrast, an increase in neutralizing antibody response induced by a booster dose against Delta^[32,48,67]^ and Omicron^[49,68]^ variants in the general population and immunocompromised patients has been reported by various authors. Immunocompromised patient populations include kidney transplant recipients^[53–61,66,68–70]^ and patients undergoing chronic hemodialysis.^[51,57,63,74,76]^

Although >90 vaccines for COVID-19 are under clinical trials, 23 vaccines have been approved for use in different countries in 2021.^[77,78]^ The main factors that could determine vaccine efficacy and duration of effectiveness include the study design, methods and sponsorship, vaccine-induced immune response, new virus variants,^[78]^ use of homologous or heterologous booster vaccinations, duration of patients’ follow-up, and transparency of the official notification of new cases. Some data showed that the Delta variant had little effect on vaccine effectiveness compared to the Omicron variant. This difference could be explained by the fact that the latter has 32 amino acid mutations in the spike protein, while there are only 15 mutations in the Delta variant.^[79]^ Moreover, 1 study showed that using different booster doses (BNT162b2, mRNA-1273, and Ad26.COV2.S) of the same developer (homologous) increased the neutralizing antibody (NAb) titers 4 to 20 times; however, boosting with different developer (heterologous) increased NAbs 6 to 76 times.^[80]^ Several studies have reported the safety of vaccine administration based on adverse events.^[15,23,41,48,57,64,71,74]^ A longitudinal observational study among healthcare workers (n = 444) reported adverse events of >75%.^[15]^ This study found that effective innate and adaptive immune responses make women more prone to adverse events. A placebo-controlled, observer-blinded, multinational, pivotal efficacy trial in the general population (n = 46,429) reported that systemic events were mostly mild to moderate in severity; however, occasional severe events occurred.^[23]^ These 2 studies aimed to establish the waning of immunity with 2 doses of the BNT162b2 vaccine (Table 1). Randomized, double-blind, placebo-controlled phase 1 and 2 trials conducted in China to assess the safety and immunogenicity of the protein subunit vaccine ZF2001 reported that the frequency of adverse events between the vaccine and placebo groups was similar in both phase 1 and phase 2.^[41]^ A prospective study evaluating the effect of a booster dose of BNT162b2 in individuals after a completed Ad5-nCoV vaccination regimen found that patients receiving BNT162b2 after Ad5-nCoV had higher SARS-CoV-2 spike 1 to 2 IgG antibody titers and no severe adverse reactions.^[47]^ In addition, a Taiwanese study revealed that most of the adverse events reported were mild and transient and were less frequent when heterologous boosting was performed at 8 weeks than at 4 weeks.^[48]^ A phase 1 trial among cancer patients in the United States reported mild adverse events associated with 3 doses of the BNT162b2 vaccine.^[64]^ Similarly, another study among liver transplant recipients and patients with chronic liver disease in the United States reported that no patient experienced any serious adverse events after 2 doses of mRNA vaccines or a single dose of the JnJ vaccine.^[71]^ According to a prospective study among hemodialysis or peritoneal dialysis patients in France, adverse events were rare and not severe after a third dose of BNT162b2.^[74]^ Table 4 summarizes the types, efficacy, and immunity of the commonly used 10 vaccines against COVID-19.^[77,78,81]^ The release of antibodies (NAbs) and activation of T cells after vaccine administration are shown in Table 4.

**Table 4:** Types, efficacy, and immunity of the commonly used vaccines against COVID-19.

Vaccine name	Mechanism of action	Efficacy, immunity, and waning
Pfizer/BioNTech	An mRNA-based vaccine, only the mRNA of the spike protein is isolated from the SARS-CoV-2 and included within a nanoparticle that is injected IM. The translation of the viral protein takes place, attracting antibodies, further producing cytokines.	Upon receiving 2 doses of the vaccine, 95% effectiveness in symptomatic COVID-19 aged 16 yr and older, 79% in asymptomatic, and 90% in hospitalized. As for more severe variants, the efficacy is 87.5%.
BNT162b2		Antigen-specific IFN-γ, CD4^+^, and CD8^+^.
		T cells and significant Nabs appear only after 2 doses.
		Vaccine’s effect starts waning soon after the peak of the second dose. it still protects from variants causing severe diseases. It is recommended by the CDC to take the booster doses every 5 mo. Protection after 2 doses decreased from 88% at 1 mo to 74% at 5 to 6 mo.
Moderna	As above	As with all mRNA-based vaccine, S-binding antibody appears after 2 wk and increases markedly after 2 doses. Nabs increased after second dose. The effectiveness and protection of the vaccine in symptomatic is 90%–94% after 2 doses.
MRNA-1273		Significant increases in CD4 + T-cell secreting.
		TH1 type cytokines after 2 doses, minimal change in TH2 cell responses and low levels of CD8^+^ responses.
		It has been reported that the vaccine’s efficacy wanes over time, but its protectiveness against severe variants still is substantial.
Novavax	An adjuvant recombinant protein vaccine, it is made up of the spike glycoproteins and adjuvant saponin to enable an immune response.	S-binding antibody detected 3 wk after first dose and markedly increase after 2 doses.
NVX-CoV2373		Nabs significantly increase 1 wk after 2 doses. CD4^+^ T-cell responses present by 1 wk after second dose.
		Initially, it was found to be as effective as 89.7% against the B.1.1.7 variant, but it dropped to 60% against the B. 1.351 variant.
Janssen/Johnson & Johnson	An engineered adenovirus 26 CoV vector vaccine, it provides a blueprint of a stable spike protein present in the coronavirus surface, which is then replicated.	S-binding and NAbs present 1 mo after vaccination in 99% and antibody levels sustained until at least 84 d. CD4 + and CD8^+^ T-cell responses present at 2 wk and 4 wk after vaccination.
Ad26.COV2.S		
		The effectiveness range is approximately 72%–65% depending on the geographic (72% USA and 57% in South Africa).
CanSino biologics	An Ad5 viral vector, a replication-incompetent vector of adenovirus that produces a spike protein eliciting an immune response.	The efficacy of the vaccine in protecting against symptomatic infections is 57.5% and around 91.7% against severe disease.
Ad5-based COVID-19 vaccine		
AstraZeneca Oxford	A viral vector with chimpanzee DNA adenovirus. It is a replication-deficient modified DNA vector. It is created in a way that does not cause a disease; however, it can generate an immune response.	S-binding antibody markedly increases after second dose. Peak T-cell responses 14 d after first dose, but slightly higher after second dose. Increase of TNF and IFN-γ release by CD4^+^ T cells after 2 wk.
ChAdOx1 nCoV-19/AZD1222		The effectiveness of the vaccine is calculated in its ability to prevent the occurrence of an infection but not the symptoms or disease. It is reportedly about 70% after the 2 doses administration.
		The efficacy wanes from 77% to 67% at the fourth to fifth month.
Gamaleya	An adenovirus recombinant using 2 replication-incompetent vectors (Ad 26 and Ad 5). Cells are infected with engineered DNA, which then replicates coronavirus spike protein to generate antibodies.	The efficacy is around 91.6% after the administration of the 2 doses.
Gam-COVID-Vac/Sputnik V		
Sinopharm	An inactivated whole virus vaccine that includes 2 SARS-CoV-2 variants has an adjuvant: aluminum hydroxide.	The efficacy in terms of reducing symptomatic infections is 94.7%, hospitalization is 60.5%, and mortality risk is 98.6% was reported after the second dose.
WIV04 and HB02		
Sinovac	An inactivated viral antigen vaccine, which generates an immune response but is weakened so it cannot cause a disease.	The efficacy reported for this vaccine is in terms of preventing COVID-19, preventing hospitalization, risk of ICU admission, and mortality by 65.9%, 87.5%, 90.3%, and 86.3%, respectively.
CoronaVac		
Bharat Biotech	An inactivated vaccine that contains an adjuvant, aluminum hydroxide, and a toll-like receptors against the adjuvant.	Anti–S-binding antibodies increase to 98%.
Covaxin/BBV152		2 wk after second dose; NAbs increase to 97% 2 wk after second dose; geometric mean titer for binding and NAbs markedly elevated by second dose.
		CD4^+^ CD45RO^+^ memory T-cell increase after 3.5 mo post second dose. The vaccine’s efficacy against preventing symptomatic infection is 78%.

CDC = centers for disease control and prevention, COVID-19 = coronavirus disease 2019, ICU, intensive care unit, IFN-γ = interferon gamma, IM = intramuscularly, mRNA = messenger ribonucleic acid, NAb = neutralizing antibody, SARS-CoV-2 = severe acute respiratory syndrome coronavirus 2, TNF = tumor necrosis factor.

## 4. Discussion

Most published studies have no long-term follow-up (only a few months) and do not answer all the questions that assure the community about the evidence-based need and safety of more vaccine shots, whether it should be from the same previously used vaccine or whether there is a need to review the mechanism of action of the booster vaccine. The short-term efficacy of a 2-dose regimen of the Pfizer BNT162b2 mRNA COVID-19 vaccine in randomized clinical trials and observational settings has been well established.[82–84] The effectiveness of a vaccine in asymptomatic or symptomatic individuals relies on multiple favorable mechanisms, such as efficient NAbs against the viral spike protein in a dose-dependent manner, activation of virus-specific CD4+ and CD8+ T cells, and release of potent immunomodulatory cytokines.[85] Although many observational studies have reported real-world data on vaccine effectiveness, reports on waning immunity or effectiveness have emerged, and the long-term efficacy of different variants remains unclear in terms of antibody dynamics over time.[60,86] In this context, 2 studies with systematic sampling of the vaccinated population reported a modest waning in vaccine effectiveness against SARS-CoV-2 infection over time.[17,21] These studies have made headlines worldwide regarding waning immunity after vaccination and have accelerated the debate on vaccine booster shots. However, the actual scenario of waning immunity is complex.

Immunological investigations have revealed a continuous reduction in antibody levels among vaccinated people.^[23]^ Long-term follow-up of vaccination trial participants has shown an increased risk of recurrent illnesses.^[87]^ A recent study included 4000 vaccinated healthcare professionals who underwent monthly antibody testing during a 6-month longitudinal prospective trial. Antibody titers decreased by 38% for IgG antibodies and 42% for neutralizing antibodies in individuals aged 65 years and older after 6 months of vaccination.^[17]^ Another report revealed that the efficiency of Pfizer BNT162b2 peaked at 78% after the second dose before progressively decreasing, with the reduction accelerating after the fourth month to approach 20% within 5 to 7 months. However, for the efficacy of this vaccine against any severe, critical, or fatal cases of COVID-19, researchers found that it peaked at approximately 96% in the first 2 months following the second dose and then remained at that level for 6 months.^[21]^ Evidence of protection against severe, critical, or fatal cases of COVID-19 beyond 6 months after the second vaccine dose has not been reported to date. In a study conducted in Israel, efficacy data were collected 14, 21, and 28 days postvaccination. The infection rates were extremely low, with an estimated efficacy of >95% for total infection, symptomatic illness, hospitalization, and mortality.^[88]^

The current vaccination efficacy data are concerning. Vaccinated people are becoming infected with SARS-CoV-2 at a higher rate than that a few months ago. According to recent centers for disease control and prevention statistics, when the Pfizer–BioNTech vaccine first became publicly available in spring, it prevented infection at a rate of up to 90%.^[89]^ Currently, this figure is at around 60% to 70%, which is still a remarkable achievement. This may exaggerate the advantages of the vaccines. Many infections are discovered merely by detecting viral genetic material, with no certainty that this material is active, infectious, or anything more than the wreckage of a successful immune defense, which is more indicative of “exposure” to the virus rather than “infection.” Two studies from the Middle East have claimed that more significant decreases are muddled and may overplay the situation.^[17,21]^ Across nations, early vaccination users were older, had somewhat worse health, and were in higher-risk professions than those who later received the vaccine. Consequently, the protection these individuals receive may be less spectacular. Furthermore, when initial effectiveness figures were established, people were still more likely to practice physical distancing and mask use.

### 4.1. SARS-CoV-2 vaccine doses: the booster appears to improve protection against infection

Several studies have been published that discussed the effects of SARS-CoV-2 vaccination boosters on human immunological responses.[32–50] However, the effect of new variants on vaccine effectiveness has not been well studied. Furthermore, the minimal level of neutralizing antibodies required to prevent infection or severe clinical consequences, as well as the relevance of vaccine-induced memory B and T cells against SARS-CoV-2, remains unknown. Therefore, the findings of these studies should be interpreted cautiously and should not be generalized to the entire human population.

Generally, a booster dose can significantly increase neutralizing antibody levels or surrogate indicators (e.g., antispike IgG) against SARS-CoV-2. A booster dose increases the level of Abs with cross-reactivity against distinct SARS-CoV-2 variants of concern (VOCs).^[32]^ These findings imply that maintaining a high level of SARS-CoV-2-specific Abs through repeated immunization is adequate to suppress COVID-19 caused by existing antigenic variations. Recent Israeli real-world data revealed that the third dose of an mRNA vaccine significantly lowered the probability of infection or severe illness caused by the delta variant.^[34–37]^ Adverse effects caused by a booster of mRNA vaccination are often moderate and equivalent to those caused by a second dose of mRNA vaccine. Currently, there is no evidence that a COVID-19 vaccination booster dose can cause unexpected adverse responses or increase the likelihood of serious adverse reactions. Although booster dosage poses no immediate risk, the long-term immunological implications of repeated vaccines are unknown. As SARS-CoV-2 continues to evolve, we still do not know whether repeated immunizations with the same antigens cause unfavorable immunological imprinting, skewing the breadth of our immune response in future encounters with other unique SARS-CoV-2 antigenic variations. Some initial studies on vaccine-induced immune responses in recovered COVID-19 individuals argue against this possibility^[39]^; however, further characterization of Ab, T-cell, and B-cell immune memory responses as well as their longevity in individuals who have received multiple doses of COVID-19 vaccines is required. Overall, existing evidence has demonstrated that booster dosages are safe and have been shown to increase the levels of SARS-CoV-2-specific neutralizing antibodies that are cross-reactive with contemporary VOCs. However, current vaccines provide effective and long-lasting protection against COVID-19–related hospitalizations and deaths across all age groups, implying that a booster dose may not be required for all fully vaccinated individuals. Therefore, the usefulness of booster dosages is still under investigation.

### 4.2. Waning immunity and need for booster vaccinations: scientific deliberations

Immunologically, IgG and neutralizing antibody titers are expected to decrease over time after vaccination. However, after mRNA vaccination, robust and long-lasting plasmablast and germinal B-cell responses have been demonstrated, and memory B cells have increased over the course of at least 6 months, improve functionally, and provide cross-variant protection.[90] Neutralizing antibody titers may suggest a degree of protection against symptomatic disease; however, there is no evidence to support this notion in the long term. Furthermore, given the documented differences in long-term efficacy against severe disease versus infection, neutralizing antibodies are unlikely to be the only players involved in the primary mechanism of protection; cellular immunity is more significant in long-term protection against severe disease.[91] The above explanation might seem incompatible with recent reports on the diminishing effectiveness of COVID-19 vaccines and waning immunity. The reality of the current Fiasco vaccine is markedly more complex.

### 4.3. Immunological correlate of protection

Another concern is whether there is an instantaneous immunological correlate of protection or a measurement of individual-level immune responses that may predict how protected an individual is against breakthrough infection at any point in time.[92–94] Neutralizing antibody titers in the first few months following vaccination appear to be strongly linked to vaccine efficacy, as evaluated in randomized trials,[91,95] and are indicative of the likelihood of breakthrough infection in individuals.[4] However, no specific antibody or neutralizing threshold titer has yet been established to predict the degree of protection as it fluctuates with waning or boosting over time. Moreover, despite the fact that the majority of studies demonstrate that adaptive immune responses induced by SARS-CoV-2 Infection exist and may protect against reinfection,[96] SARS-CoV-2 experience suggests that immunity to natural infection may diminish with time, and reinfection has been reported.[97] Clearly, it is critical to conduct studies to investigate the link between immune response parameters and the risk of reinfection. Furthermore, vaccine effectiveness data for booster doses are being released from a growing number of countries; however, follow-up time remains limited. All trials showed improvements in protection against infection, mild disease, severe disease, and death. Additional booster doses may be required to increase the period of protection; however, it is unclear whether the initial 2 doses and booster doses are different. It is also unknown whether previously infected people will benefit from vaccination; nonetheless, at this stage, immunization against SARS-CoV-2 should occur regardless of the infection status. The geometric mean titers (GMT) of NAbs have been shown to increase in a dose-dependent manner 3 weeks after the priming vaccine dose and go higher 1 week after the booster dose.[85] The role of cellular T-cell responses in vaccine-mediated protection against COVID-19 is of utmost importance, although few studies have addressed this. The T-cell response is needed to support the production and to maintain high-affinity antibodies.[98] The virus-specific CD4 + response peaks after the booster dose of BNT162b2 fell to the prebooster level after 4 months. However, adenovirus-based vaccines induce stronger responses than mRNA-based vaccines.[98] The latter produces higher antibody titers, a finding that encourages the use of heterologous vaccination.

### 4.4. Breakthrough infections and randomized controlled clinical trials

At the time of writing, a key public health topic is whether vaccination protection diminishes over time or as new virus strains circulate, increasing the probability of breakthrough infections, given a certain degree of exposure. Although real-world evidence on antibody kinetics is emerging, a comprehensive picture of the duration of immunity is lacking. Long-term vaccine effectiveness evaluation in phase III randomized controlled trials (RCTs) has been limited because vaccine randomized efficacy studies administered immunization to placebo users soon after vaccines were approved for emergency use.[99] Nonetheless, such data were available for up to 6 months after the initial dose,[23,100,101] and breakthroughs were more common among earlier-vaccinated patients, providing evidence for declining vaccine effectiveness. Furthermore, current phase III RCTs have been designed to identify protection against symptomatic infections. However, protection against severe COVID-19 and death is the primary objective of implementing a COVID-19 vaccine, and knowledge of vaccination protection against COVID-19 may not allow the prediction of protection against severe disease. Another drawback of RCT data is that they can only accurately predict protection against a single viral variant rather than comparing protection across variants. Phase III trials were conducted, in which 1 variant was dominant in each nation during the study period. As a result, the greater incidence of breakthrough infections in a nation with a specific variant could not be attributed to a certain variant since other factors also differed between countries.[102] Furthermore, these trials tested vaccination with a placebo, eliminating the possibility of head-to-head comparisons of many vaccines in an RCT setting.[103,104] Therefore, RCT designs might overestimate the level of vaccine protection compared with real-world settings. These dynamic estimates from different studies and countries are significantly affected by the prevalence, behavior, and circulation of different variants, making comparisons unreliable for evaluating changes in immune protection over time. Therefore, the immunological argument for a COVID-19 booster in this early stage remains unclear.

The third dose of the vaccines produced by Pfizer–BioNTech and Oxford–AstraZeneca, administered >6 months after immunization, may increase neutralizing antibody titers, including against the delta variant. Furthermore, data from these studies indicate that vaccine-related adverse effects were comparable to those found following the first and second doses of vaccinations.^[32,60,105]^ Therefore, it seems prudent to identify the most susceptible population that may benefit from a third dose without risking the worldwide COVID-19 immunization campaign. The safety profile and possible additional protection of a third dose must be evaluated against the vaccine’s global scarcity.

### 4.5. Enormous data, yet a slew of unanswered questions

The distribution of COVID-19 vaccines has occurred with remarkable speed and attention in nations with substantial supplies, possibly best highlighted by the fact that several studies have provided an assessment of vaccination efficacy on each particular day following vaccination.[37] Simultaneously, precise antibody kinetics have been assessed in hundreds of individuals, providing information on the temporal patterns of immune responses.[17,33,87] In many countries, the right balance between nonpharmaceutical therapies and booster dose vaccination programs in addressing delta variants and Omicron-driven surges is fiercely debated.[106–108] Some countries that discontinued all nonpharmaceutical interventions after attaining high levels of vaccine coverage were forced to reintroduce these measures in the face of massive resurgence[16] while implementing a population-wide third-dose mass-vaccination campaign to avoid the need for further restrictions.[37] As more variants of VOCs are likely to emerge and more countries experience waning immunity, these arguments are likely to escalate in light of worldwide constraints on vaccine supplies for primary immunization, which are particularly acute in low-income and lower-middle-income countries.[8] Many factors must be considered before a booster can be offered. A country may decide to administer a booster dose in selected segments of the population depending on epidemiological circumstances, vaccination coverage, and population immunity owing to infection-induced immunity. Moreover, even if some gain can be obtained from boosting, it does not outweigh the benefits of providing initial protection to unvaccinated individuals. Ideally, focusing on vaccinating unvaccinated individuals would yield greater benefits. The World has advocated a moratorium on boosting until the advantages of primary vaccination are made available to a greater number of individuals worldwide.[109]

Furthermore, it is unclear whether the SARS-CoV-2 vaccine developed using strains recovered at the start of the pandemic in 2019 and 2020 will protect against emerging VOC circulating in 2021 and 2022. Another question is whether a third dose administered months after the second will be qualitatively different from the second and provide higher long-term protection against breakthrough infections or whether protection levels would quickly revert to preboost levels.^[110]^ The enormous epidemic surge in the postvaccination era demonstrates the capacity of transmissible genotypes to defy COVID-19 control even in areas with high coverage, which is presently a more serious hazard than dwindling immunity. The study of breakthrough infections in clinical trials may provide insights into the variations in neutralization resistance. If neutralizing antibodies are proven to be protective, newer COVID-19 vaccines may be required. A global surveillance program could be used to track changes in circulating SARS-CoV-2, which could help to develop new vaccines more quickly in the future. Increasing data on the efficacy of booster immunizations have worldwide ramifications, as booster recommendations are likely to result in increased supply being diverted to high-income countries. Sufficient vaccine supplies must be made available to guarantee a high degree of protection against SARS-CoV-2 infection. Failure to do so perpetuates the conditions that encourage the emergence of new VOCs.

Lastly, as the vaccination for every variant is not practical or feasible, progress in the field of bioinformatics allows methods to fight COVID-19 and predict new variants through the use of in silico models for phylogenetic analysis, peptidomimetics, and SARS-CoV-2 vaccine design.^[111]^

### 4.6. Limitations

Limitations of this quick scoping review include the lack of sufficient time, risk of bias assessment (critical appraisal) is not applicable, and the review may have missed some relevant studies as we did not include gray literature search and non-English language articles.

## 5. Conclusion

We believe that using laboratory indicators over real-world indicators to rationalize the expanded indications for additional vaccine doses lacks strong scientific evidence. The rush to mandate a third or more vaccination dose based on findings from interim data that originated from short follow-up periods lacks evidence of long-term safety and efficacy. There has been an increase in the infection rate among the populations of countries in which 2 doses and even 3 doses of the vaccine have been administered, but with less severity. Most of these infections are related to new variants, a finding that may require revision of the boosting vaccine or more time. The implementation of booster vaccinations must be wielded efficiently and in tandem with other evidence-based public health interventions. A comprehensive preventative program, more studies on vaccine optimization to expand its effectiveness against new viral variants, correlates of protection, long-term safety, and ongoing surveillance are required, in addition to consistent implementation of vaccination programs. Adequate scientific information and public awareness are required regarding the administration of booster doses to the general population as well as the high-risk individuals.

## Author contributions

All authors have substantial contribution, read and approved this manuscript.
